# All-optics technique for monitoring absolute cerebral blood flow: validation against magnetic resonance imaging perfusion

**DOI:** 10.1117/1.NPh.11.4.045002

**Published:** 2024-10-03

**Authors:** Leena N. Shoemaker, Saeed Samaei, Graham Deller, Danny J. J. Wang, Daniel Milej, Keith St. Lawrence

**Affiliations:** a Western University, Department of Medical Biophysics, London, Ontario, Canada; b Lawson Health Research Institute, Imaging Program, London, Ontario, Canada; c Western University, School of Kinesiology, London, Ontario, Canada; d University of Southern California, Mark and Mary Stevens Neuroimaging and Informatics Institute, Keck School of Medicine, Laboratory of fMRI Technology, Los Angeles, California, United States

**Keywords:** diffuse correlation spectroscopy, near-infrared spectroscopy, arterial spin labeling, hypoxia, cerebral blood flow, hypercapnia

## Abstract

**Significance:**

The ability to monitor cerebral blood flow (CBF) at the bedside is essential to managing critical-care patients with neurological emergencies. Diffuse correlation spectroscopy (DCS) is ideal because it is non-invasive, portable, and inexpensive. We investigated a near-infrared spectroscopy (NIRS) approach for converting DCS measurements into physiological units of blood flow.

**Aim:**

Using magnetic resonance imaging perfusion as a reference, we investigated the accuracy of absolute CBF measurements from a bolus-tracking NIRS method that used transient hypoxia as a flow tracer and hypercapnia-induced increases in CBF measured by DCS.

**Approach:**

Twelve participants (7 female, 28±6 years) completed a hypercapnia protocol with simultaneous CBF recordings from DCS and arterial spin labeling (ASL). Nine participants completed the transient hypoxia protocol while instrumented with time-resolved NIRS. The estimate of baseline CBF was subsequently used to calibrate hypercapnic DCS data.

**Results:**

Moderately strong correlations at baseline (slope=0.79 and $R^{2}=0.59$) and during hypercapnia (slope=0.90 and $R^{2}=0.58$) were found between CBF values from calibrated DCS and ASL (range 34 to 85  mL/100  g/min).

**Conclusions:**

Results demonstrated the feasibility of an all-optics approach that can both quantify CBF and perform continuous perfusion monitoring.

## 1 Introduction

Worldwide, acquired brain injuries—ischemic stroke, severe traumatic brain injury, and subarachnoid hemorrhage—are major killers and extremely resource-demanding in terms of direct healthcare costs and long-term disability.^1^ Intensive care of patients with life-threatening acquired brain injuries not only saves lives but improves neurological outcomes. A primary focus is preventing delayed cerebral ischemia by treating complications that can impede oxygen delivery to the brain, such as systemic hypotension and elevated intracranial pressure.^2^ Clinical features related to neurological deficits (e.g., paresis) are used to detect worsening neurological status, but these often manifest after ischemic injury has occurred and are not reliable indicators in patients requiring sedation. Studies using invasive probes capable of quantifying cerebral blood flow (CBF), such as thermal diffusion flowmetry, suggested that CBF monitoring improves outcomes by alerting the intensivist team when CBF falls below ischemic thresholds.^3^ The invasiveness of such probes has hindered greater adoption and speaks to the need to develop non-invasive alternatives.

Diffuse correlation spectroscopy (DCS) is rapidly emerging as a non-invasive optical technology capable of continuously monitoring CBF.^4^^,^^5^ Multiple animal model studies have demonstrated that the blood flow index (BFi) obtained with DCS strongly correlates with CBF data from other techniques.^6^ The cerebral metabolic rate of oxygen can also be measured by combining BFi with tissue oxygen saturation (${\mathrm{StO}}_{2}$) measurements from near-infrared spectroscopy (NIRS).^7^^,^^8^ DCS in combination with various types of NIRS devices has been used to monitor CBF in various patient populations requiring intensive care, ranging from preterm infants to adult patients with acute brain injuries.^9^^–^^13^ Most of these applications used DCS as a trend monitor to investigate the stability of relative CBF. Measuring absolute CBF would enhance clinical applications by enabling longitudinal monitoring to compare day-to-day changes in CBF. However, obtaining BFi estimates that accurately reflect absolute CBF is challenging as it requires modeling light propagation through the structures of the adult head and accounting for blood flow in the scalp and brain.^14^^–^^17^

An alternative approach is to measure absolute CBF by dynamic contrast-enhanced (DCE) NIRS and use this value to convert relative BFi data into a time series in physiological units of blood flow (i.e., mL of blood /100 g of tissue/min^18^^–^^20^). DCE NIRS uses a light-absorbing dye, indocyanine green (ICG), as an intravascular contrast agent and has good agreement with CBF estimates from the magnetic resonance imaging (MRI)-based perfusion method arterial spin labeling (ASL).^21^ Accurate CBF measurements by DCE NIRS were accomplished in part using time-resolved NIRS (trNIRS) to enhance depth sensitivity. The ability to convert BFi to standard flow units provides a means of comparing DCS results to CBF data from other perfusion methods and determining if CBF estimates measured at the bedside have fallen below ischemic thresholds. Although ICG is a relatively nontoxic dye used in ophthalmology and surgical procedures,^22^ the use of an exogenous contrast agent adds complexity to perfusion monitoring with DCS as it requires an intravenous catheter and following safety procedures in case of an adverse event.

The purpose of this study was twofold. The first aim was to investigate an alternative DCE NIRS approach for calibrating DCS using a brief reduction in arterial oxygen saturation (${\mathrm{SaO}}_{2}$) as a flow tracer (i.e., transient hypoxia). Manipulating ${\mathrm{SaO}}_{2}$ was the first method proposed for measuring CBF with NIRS; however, the signal-to-noise ratio (SNR) of the original method was poor due to the required short measurement duration.^23^ In the current study, this limitation was avoided using a kinetic modeling approach to analyze dynamic data^24^—an approach referred to as dynamic hypoxia contrast (DHC) NIRS. This approach is similar to recent protocols developed for blood oxygen level-dependent MRI.^25^^,^^26^ Using a hybrid trNIRS/DCS system, the second aim was to investigate the ability of the DHC-NIRS/DCS combination to accurately measure resting CBF and the increase in CBF caused by hypercapnia.^21^^,^^27^^–^^29^ DCS and ASL data were acquired simultaneously to compare hypercapnic CBF responses. Baseline CBF was measured by DHC-NIRS immediately after the MRI session when participants had returned to normocapnia.

## 2 Methods

### 2.1 Participants

All procedures were approved by the Health Sciences Research Ethics Board (HSREB) of Western University (No. 105417) and conducted in accordance with the Declaration of Helsinki ethical standards. Participants provided written informed consent following verbal and written explanations of the experimental procedures. Thirteen healthy participants were recruited. Before instrumentation, participants completed a mandatory health history form to evaluate the inclusion/exclusion criteria. Participants were included if they passed the MRI screen form and reported being nonsmokers with no prior diagnosis of cardiovascular disease, neurological disorder, diabetes, or hypertension. All subjects reported that they identify with their sex assigned at birth.

One male participant was excluded from all analysis as a result of equipment difficulties. Therefore, a total of twelve participants (n=12) were subsequently included in the ASL and DCS hypercapnia analysis. A subset of participants (n=9; 4 female) completed two additional protocols (DHC-NIRS and hypercapnia with trNIRS) described below.

### 2.2 Instrumentation

Participants breathed through a mask sealed to the face with skin tape (Tegaderm, 3M, Saint Paul, MN) to prevent air leakage. The facemask was connected to a computerized sequential gas delivery system (RespirAct™, Thornhill Medical, Toronto, Canada) that controlled end-tidal partial pressure of oxygen ($P_{\mathrm{ET}}O_{2}$) and carbon dioxide ($P_{\mathrm{ET}}{\mathrm{CO}}_{2}$) independent of the pattern of participant ventilation.

A custom-designed optode holder was placed on the subject’s right lateral forehead and secured with a Velcro headband. Importantly, the headband was tightened to reduce extracerebral blood flow contamination with optical measures.^30^^–^^32^

The study utilized a custom-built hybrid system that combined trNIRS and DCS instruments, which is described in detail elsewhere.^28^^,^^33^^–^^35^ Briefly, the trNIRS module used two pulsed lasers (repetition rate = 80 MHz) operating at 760 and 832 nm (PicoQuant, Berlin, Germany). Light pulses from the laser heads were coupled into a multimode bifurcated fiber (Φ=200  μm, NA=0.22, Loptek, Germany). Diffusely reflected light was collected at a source-detector separation ($r_{\mathrm{SD}}$) of 3 cm and delivered to a hybrid photomultiplier tube (PMA Hybrid 50, PicoQuant, Berlin, Germany) via a multimodal fiber (Φ=400  μm, NA=0.39, Loptek, Germany). A time-correlated single-photon counting unit (HydraHarp 400, PicoQuant, Berlin, Germany) was used to record photon arrival times and build distributions of time-of-flight (DTOFs^36^). To enable simultaneous trNIRS/DCS acquisition, a short-pass interference filter (Spec 3551, 836.5 nm, diameter 25 mm, Alluxa, Santa Rosa, California, United States) was placed in front of the trNIRS detector. Finally, the system instrument response function (IRF) was measured at the end of each experiment using a custom light-tight box.

The DCS module employed a long coherence length laser operating at 852 nm (CrystaLaser, Reno, Nevada, United States). The laser light was delivered to the tissue through a multimode fiber (Φ=400  μm, NA=0.39, FT400UMT, Thorlabs, Newton, New Jersey, United States). The diffusively reflected light was collected by three single-mode fibers (Φ=4.4  nm, NA=0.13, 780HP, Operating Wavelength = 780 to 970 nm, Thorlabs) at $r_{\mathrm{SD}}=2.7\;\mathrm{cm}$, which were coupled to a four-channel single photon counting module (SPCM-AQR-15-FC, Excelitas Technologies, Montreal, QC, Canada). The output from each detector was fed into an edge-detecting counter on a PCIe-6612 counter/timer data acquisition board (National Instruments, Austin, Texas, United States). Photon counts were recorded and processed using custom software (LabVIEW, National Instrument, Austin, Texas, United States^37^) that generated intensity autocorrelation curves for 50 delay times (τ) ranging from 1  μs to 1 ms.^38^ Time-resolved NIRS and DCS data were acquired at a sampling frequency of 3 Hz in all protocols.

### 2.3 Experimental Design

After obtaining informed consent and completing instrumentation (described above), participants provided a venous blood sample (1 mL) from the antecubital vein to assess baseline hematocrit concentration (ABL80 Flex Co-ox, Radiometer, Copenhagen, Denmark). All protocols consisted of participants resting in a supine posture. The headband and optode holder were not moved between protocols. Briefly, participants completed three protocols, listed in order of occurrence: (1) ASL hypercapnia protocol, (2) dynamic hypoxia contrast (DHC) protocol, and (3) trNIRS hypercapnia protocol.

### 2.4 MRI Acquisition

Hypercapnia experiments were performed using a 3T Biograph mMR scanner (Siemens Medical Systems, DE, Erlangen, Germany) with a 12-channel receive-only head coil. The head was immobilized with foam padding to minimize motion artifacts, and MRI fiducial marks were placed on the forehead to identify the location of the optodes on structural MRIs.

Sagittal T1-weighted images were acquired using a 3D magnetization-prepared rapid gradient echo sequence [MPRAGE; repetition time (TR) = 2 s, echo time (TE) = 2.98 ms, inversion time = 900 ms, flip angle = 9 deg, field of view (FOV)=176×256×256  mm, and isotropic $\text{voxel size}=1.0\;{\mathrm{mm}}^{3}$]. CBF images were obtained using a pseudo-continuous arterial spin labeling (pCASL) sequence with 3D segmented gradient and spin-echo readout that incorporated background suppression (voxel size $3.8\times 3.8\times 3.8\;{\mathrm{mm}}^{3}$; 26 slices; TR = 4.22 s; TE = 39.76 ms; single post labeling delay = 1.7 s; labeling duration =1.5 s; FOV=240×240×99  mm).^39^ A total of 48 pairs of tagged and control images were obtained at rest and during hypercapnia for each subject, along with a proton density-weighted image M0 (no background suppression, TR = 10 s).

### 2.5 ASL and trNIRS Hypercapnia Protocols

The ASL hypercapnia protocol consisted of 800 s (∼13  min) of simultaneous pCASL and DCS data acquisition. Datasets were collected during 409 s of normocapnic baseline, 323 s of hypercapnia (step increase of +10  mmHg), and 68 s of recovery. Cerebrovascular reactivity to ${\mathrm{CO}}_{2}$ (${\mathrm{CVR}}_{\mathrm{CO}2}$) was calculated as $\Delta {\mathrm{CBF}}_{\mathrm{ASL}}/\Delta P_{\mathrm{ET}}{\mathrm{CO}}_{2}$ (mL/100 g/min/mmHg). Upon completion of the MRI hypercapnia protocol, participants were transferred from the MRI to a hospital bed (∼3  m walking distance) and given 10 min of quiet, supine rest. Without moving the optode holder, the DCS fibers were replaced with the trNIRS fibers. After completion of the DHC protocol (described below), participants were given 10 min of wash-out prior to starting the trNIRS hypercapnia protocol. The trNIRS hypercapnia protocol consisted of a 60-s normocapnic baseline, 5 min of hypercapnia (step increase of +10  mmHg), and 120 s of recovery. Importantly, $P_{\mathrm{ET}}{\mathrm{CO}}_{2}$ was controlled by the RespirAct™ to ensure repeatability between protocols.

### 2.6 Dynamic Hypoxia Contrast Protocol

**Fig. 1:**
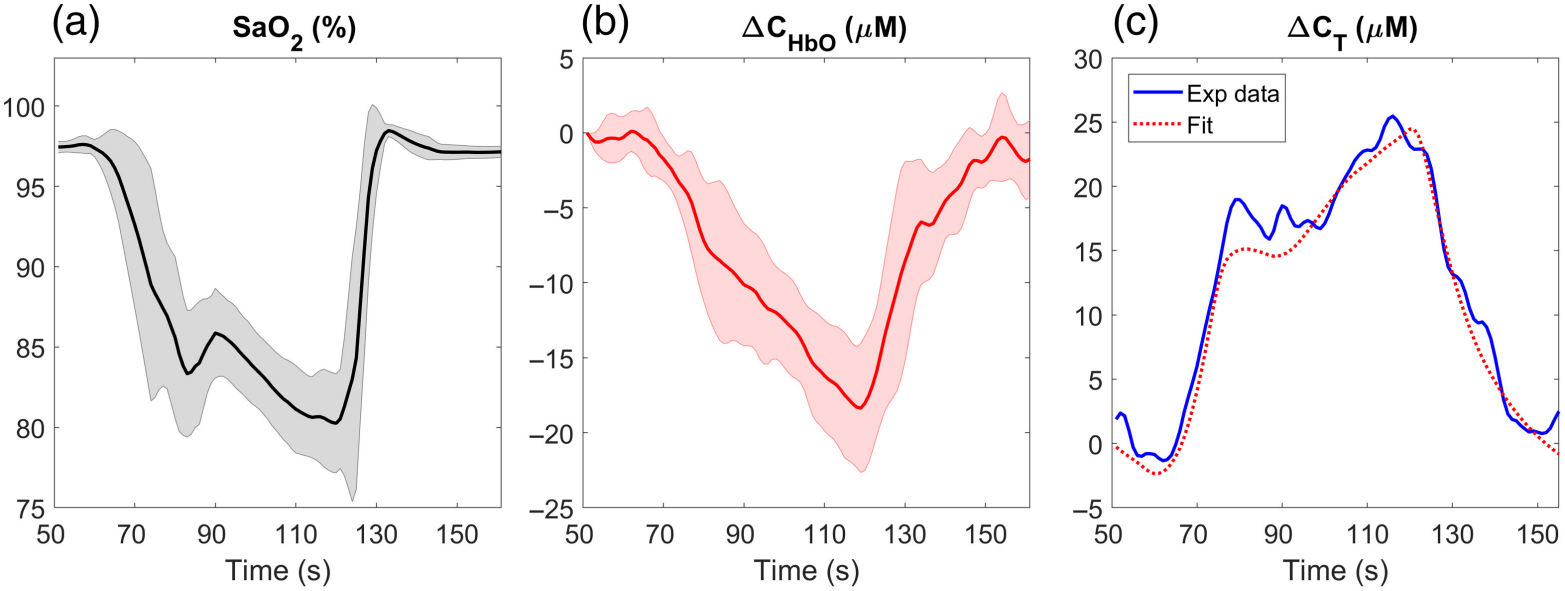
Average time courses of (a) arterial oxygen saturation (${\mathrm{SaO}}_{2}$) and (b) change in oxyhemoglobin concentration in the brain ($\Delta C_{\mathrm{HbO}}$) (n=9). Shading around each line represents the standard deviation. (c) $\Delta C_{T}$ time series of experimental data from a representative participant, along with the best fit of the perfusion model [Eq. (3)].

The DHC protocol occurred under the same conditions as discussed above and immediately prior to the trNIRS hypercapnia protocol. This programmed hypoxia protocol was 180 s long (e.g., Fig. 1), consisting of a 60-s baseline $P_{\mathrm{ET}}O_{2}$ of 95 mmHg (normoxia), a step decrease in $P_{\mathrm{ET}}O_{2}$ to 40 mmHg (hypoxia) for 60 s, and a return to normoxia for the remaining 60 s.

## 3 Data Analysis

### 3.1 MRI Image Analysis

Analysis was conducted with in-house Matrix Laboratory (MATLAB) scripts. Using the Statistical Parametric Mapping software package (SPM12),^40^ MPRAGE images were segmented into tissue probability maps for grey and white matter, cerebrospinal fluid, and non-brain matter. These images were co-registered to the pCASL M0 image.

All pCASL images were realigned to M0 for motion correction. Perfusion-weighted images were generated by averaging the pairwise subtractions of sequential tag and control images, then calibrated to M0 for CBF quantification using the equation from Ref. 39.

$$\mathrm{CBF}=\frac{6000\cdot \lambda \cdot \Delta M\cdot e^{\mathrm{PLD}/T_{1b}}}{2\cdot \alpha \cdot T1b\cdot M0\cdot (1-e^{-\mathrm{LD}/T_{1b}})}[\frac{\mathrm{mL}}{100\;g\cdot \min}],$$ (1)

where λ is the partition coefficient of water (0.9  mL/g), ΔM is the perfusion-weighted signal, PLD is the post-labeling delay (1.7 s), $T_{1b}$ is the T1 relaxation time of blood (∼1.65  s), α is the blood-labeling efficiency (0.85), and LD is the labeling duration (1.5 s). All images were smoothed with a 6-mm Gaussian filter and normalized to the Montreal Neurological Institute brain atlas.

Finally, each participant’s MPRAGE was used to measure the extracerebral layer thickness (ImageJ 1.54d). Five measurements of each layer thickness were made directly posterior to the DCS fiducial marker and averaged to yield mean skull and scalp layer thicknesses for each participant.

### 3.2 DCS Analysis

Using the Siegert relation, each normalized intensity autocorrelation function ($g_{2}$) was converted to the corresponding electric field autocorrelation function and fit with the solution to the diffusion approximation for a semi-infinite homogenous medium.^14^ The fitting incorporated subject-specific optical coefficients obtained by trNIRS and the coherence factor (β) determined from the average initial value of the baseline $g_{2}$ curve. The fitting procedure was performed for the initial part of the intensity autocorrelation curve, i.e., $g_{2}(\tau )>1.25$, to increase the sensitivity to the brain. The fitting procedure yielded a best-fit blood flow index (BFi) estimate based on modeling tissue perfusion as pseudo-Brownian motion.^6^ The resulting BFi time courses were smoothed with the zero-phase digital filter (filtfilt, MATLAB, 2016b, MathWorks, Natick, Massachusetts, United States).

### 3.3 Time-Resolved NIRS Analysis

Baseline subject-specific optical properties at each wavelength were determined from a DTOF averaged across the first 30 s of data. The mean DTOF was fit with the solution to the diffusion equation for a semi-infinite homogeneous medium convolved with the measured IRF (fminsearch, MATLAB, Mathworks Inc., Natick, Massachusetts, United States).^33^ The fitting parameters were the absorption coefficient ($\mu_{a}$), the reduced scattering coefficient ($\mu_{s}^{\prime }$), and an amplitude factor that accounts for laser power, detection gain, and coupling efficiency. The fitting range was set to 10% of the peak value of a DTOF on the leading edge and 5% on the falling edge.^29^

The first three statistical moments (number of photons N, mean time of flight ⟨t⟩, and variance V) were used to obtain depth sensitivity from the trNIRS data. The three moments were calculated for each DTOF in a time series recorded at either 760 or 832 nm by setting the lower and upper integration limits based on arrival times corresponding to 1% of the peak of the DTOF. The change in each moment relative to its initial value (mean value across 30 s at baseline) was calculated to generate three time series (i.e., ΔN, Δ⟨t⟩, and ΔV), with ΔN being more representative of extracerebral tissue and ΔV being more representative of cerebral tissue. The time courses determined for each moment were converted into absorption changes $\Delta \mu_{a}(\lambda )$ using sensitivity analysis, as described previously.^29^ The $\Delta \mu_{a}(\lambda )$ time courses for 760 and 832 nm were converted to changes in concentration of oxyhemoglobin ($\Delta C_{\mathrm{HbO}}$) and deoxy-hemoglobin ($\Delta C_{\mathrm{Hb}}$) using wavelength-specific molar extinction coefficients.^41^

### 3.4 Dynamic Hypoxia Contrast Analysis

As $P_{\mathrm{ET}}O_{2}$ is a valid index of arterial partial pressures of $O_{2}$,^25^ the $P_{\mathrm{ET}}O_{2}$ time course recorded by the RespirAct was converted to ${\mathrm{SaO}}_{2}$ using the Hill equation to describe the *in vivo* oxyhemoglobin dissociation curve.^42^ Arterial concentration of oxy-hemoglobin, $C_{a}(t)$, was calculated from the change in ${\mathrm{SaO}}_{2}$, denoted $\Delta S_{a}O_{2}$, and subject-specific total hemoglobin concentration, tHb, using the following equation:

$$C_{a}(t)=\frac{\Delta S_{a}O_{2}\cdot tHb\cdot H_{r}}{MW_{Hb}}\cdot 10^{5},$$ (2)

where ${\mathrm{MW}}_{\mathrm{Hb}}$ is the hemoglobin molecular weight (64,500  g/mol), $H_{r}$ is the large-to-small vessel hematocrit ratio (0.7), and $C_{a}(t)$ is in units of μmol/L.

Baseline CBF (${\mathrm{CBF}}_{\mathrm{DHC}}$) was estimated by modeling the relationship between $C_{a}(t)$ and the corresponding time-varying change in tissue hemoglobin concentration, $C_{T}(t)$, as a linear time-invariant system assuming a constant blood flow:^21^

$$\Delta C_{T}(t)={\mathrm{CBF}}_{\mathrm{DHC}}\cdot \int_{0}^{t}C_{a}(u)\cdot R(t-u)du,$$ (3)

where $C_{T}(t)$ was defined by the magnitude of $\Delta C_{\text{HbO}}$ derived from Δ⟨t⟩. The mean time of flight was previously found to provide a good compromise between greater depth sensitivity and SNR.^21^ R(t) is the impulse residue function and was modeled by a gamma function to characterize the distribution of capillary transit times in the tissue region interrogated by NIRS:^24^

$$R(t)=1-\int_{0}^{t}\frac{u^{\alpha -1}\cdot e^{u/\tau }}{\tau^{\alpha }\cdot \Gamma (\alpha )}du,$$ (4)

where $\Gamma (\alpha )=\int_{0}^{\infty }t^{\alpha -1}\cdot e^{-t}dt$, α characterizes the width of the distribution ($0\leq \alpha^{-1}\leq 1$), and $\tau =t_{c}/\alpha$, with $t_{c}$ representing the mean capillary transit time. A nonlinear optimization routine MATLAB^®^ function (fminsearch) was used to fit Eq. (3) to $\Delta C_{\mathrm{HbO}}(t)$ to extract best-fit estimates of the three fitting parameters, ${\mathrm{CBF}}_{\mathrm{DHC}}$, α, and $t_{c}$. ${\mathrm{CBF}}_{\mathrm{DHC}}$ was subsequently used to convert the hypercapnia BFi time series to units of blood flow (i.e., ${\mathrm{CBF}}_{\mathrm{DCS}}$)

$${\mathrm{CBF}}_{\mathrm{DCS}}(t)={\mathrm{CBF}}_{\mathrm{DHC}}(1+\Delta \text{BFi}(t)),$$ (5)

where $\Delta \mathrm{BF}\text{i}(t)=(\mathrm{BF}\text{i}(t)-{\mathrm{BF}\text{i}}_{o})/{\mathrm{BF}\text{i}}_{o}$, and ${\mathrm{BF}\text{i}}_{o}$ is the average baseline BFi value.

### 3.5 Statistical Analysis

Individual normocapnic and hypercapnic values were calculated as the average value from a ≥2-min steady-state response profile. All data are presented as mean ± standard deviation unless otherwise noted. Statistical significance was defined as p<0.05. All datasets were found to conform to a normal distribution based on visual inspection of Q-Q plots and the results of the Shapiro-Wilk test (i.e., all p≥0.22). Sex differences between normocapnic ${\mathrm{CBF}}_{\mathrm{ASL}}$ and ${\mathrm{CVR}}_{\mathrm{CO}2}$ were assessed using two-tailed t-tests. Paired t-tests were used to assess ${\mathrm{CBF}}_{\mathrm{ASL}}$ differences between normo- and hypercapnia. A two-way analysis of variance was used to compare moments (3 levels: ΔN, Δ⟨t⟩, and ΔV) across time (16 levels: 30-s bins) with post hoc testing completed using Dunnett’s test for multiple comparisons (GraphPad Prism version 10.1.2). We assessed the average $\Delta \mu_{a}$ from 832 nm (Δ⟨t⟩) as the difference between steady-state hypercapnia (minimum 2 min) and baseline values.

To examine the correlation between two techniques of deriving CBF, linear regression analysis (i.e., $R^{2}$ and r) was performed for the following data: [1] ${\mathrm{CBF}}_{\mathrm{DHC}}$ and ${\mathrm{CBF}}_{\mathrm{ASL}}$ (mL/100  g/min), [2] $\Delta {\mathrm{CBF}}_{\mathrm{DCS}}$ and $\Delta {\mathrm{CBF}}_{\mathrm{ASL}}$ (%), and [3] ${\mathrm{CBF}}_{\mathrm{DCS}}$ and ${\mathrm{CBF}}_{\mathrm{ASL}}$ (mL/100  g/min). The slope for each regression was statistically compared to a slope of 1. In addition, Bland–Altman analysis was conducted to assess the similarity between the two sets of CBF measurements.

## 4 Results

Twelve participants (7 women, 28±6 years, 72±18  kg, 174±8  cm) completed the ASL hypercapnia protocol. The average scalp thickness is 7.2±1  mm, skull thickness is 8.2±1.4  cm, and total depth to the brain (skull + scalp) is 16.4±2.7  mm (n=12). Baseline ${\mathrm{CBF}}_{\mathrm{ASL}}$ (grey matter) was 49±8  mL/100  g/min, with females having significantly higher baseline perfusion than males (54±5 versus 43±7  mL/100  g/min; p=0.03).

Of the 12 participants that completed the ASL protocol, nine participants (5 women, 29±6 years, 75±19  kg, 176±7  cm) also completed the DHC-NIRS and NIRS-Hypercapnia protocols. The average hematocrit was 46±5% (total hemoglobin: 15±2  g/dL; n=9). Females (n=4) had lower hematocrit (42±1 versus 50±4%) and total hemoglobin (14±0 versus 16±1  g/dL) than males (n=5; both p<0.01).

### 4.1 Absolute CBF from DHC-NIRS

The average reduction in ${\mathrm{SaO}}_{2}$ during transient hypoxia and the corresponding average time course of change in oxyhemoglobin concentration are shown in Fig. 1. The former was obtained from the change in $P_{\mathrm{ET}}O_{2}$ measured by the RespirAct and the latter from Δ⟨t⟩. Included in the figure is a representative case showing the perfusion model [Eq. (3)] fit to oxyhemoglobin time course from one subject.

**Fig. 2:**
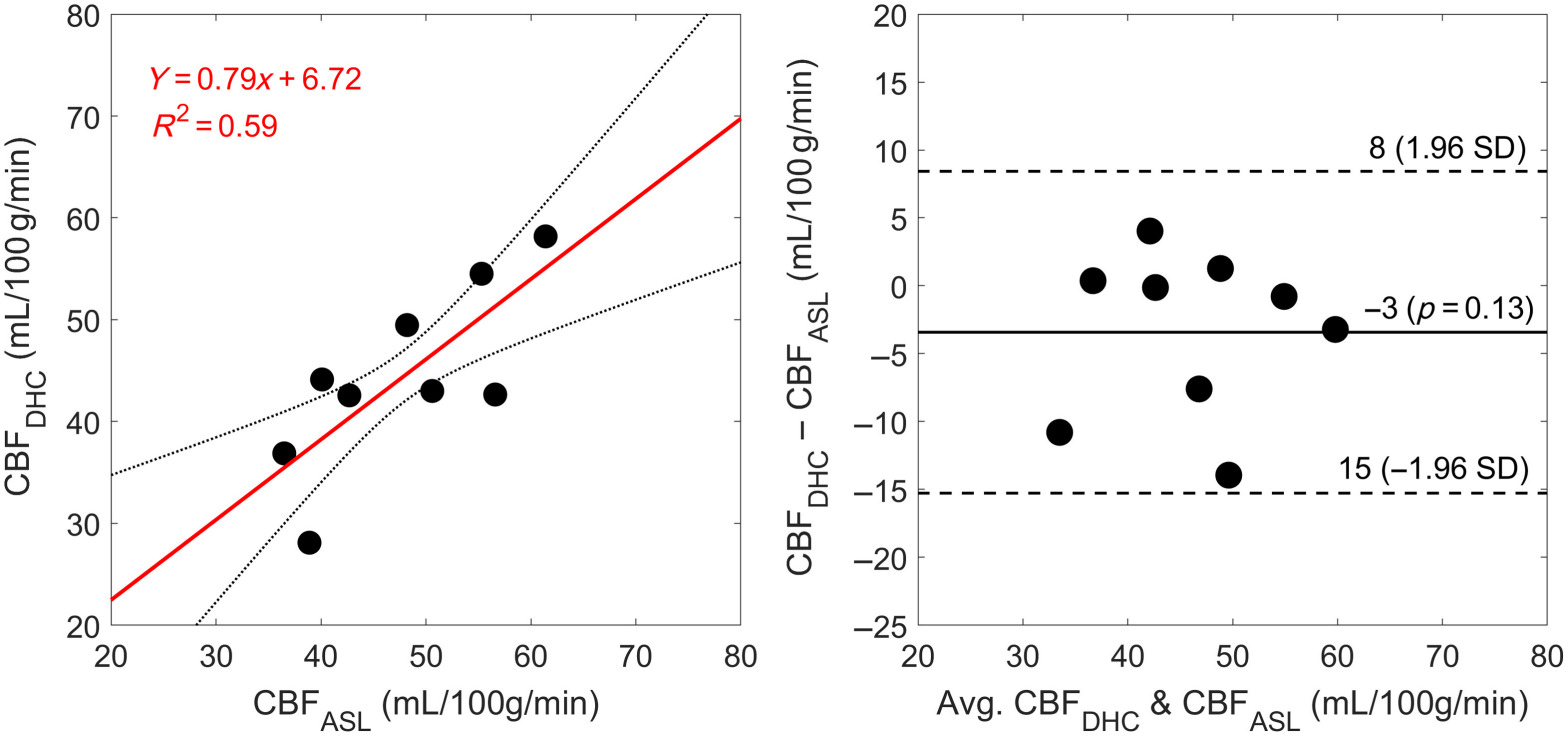
Regression and Bland–Altman plots comparing baseline CBF measures from arterial spin labeling CBF (${\mathrm{CBF}}_{\mathrm{ASL}}$) and dynamic hypoxia contrast time-resolved NIRS (${\mathrm{CBF}}_{\mathrm{DHC}}$) (n=9). The mean difference between the two methods is indicated by the solid black line, which was bound by a 95% confidence interval indicated by the dashed black lines.

Average ${\mathrm{CBF}}_{\mathrm{DHC}}$ was 44±9  mL/100  g/min (n=9) and $t_{c}$ was 8.49±3.10s. There was a strong positive correlation between absolute baseline ${\mathrm{CBF}}_{\mathrm{ASL}}$ and ${\mathrm{CBF}}_{\mathrm{DHC}}$ (r=0.77, p=0.02; Fig. 2), with a slope of 0.79 that was not significantly different than 1.00 (p=0.34) and an intercept not significantly different than 0.00 (p=0.06). However, ${\mathrm{CBF}}_{\mathrm{DHC}}$ slightly underestimated ${\mathrm{CBF}}_{\mathrm{ASL}}$, as demonstrated by a mean bias of −3  mL/100  g/min with moderately wide limits of agreement [95% CI: −15, 8] and no evident trend or inconsistent variability.

### 4.2 CBF_*ASL*_ during ASL Hypercapnia Protocol

**Fig. 3:**
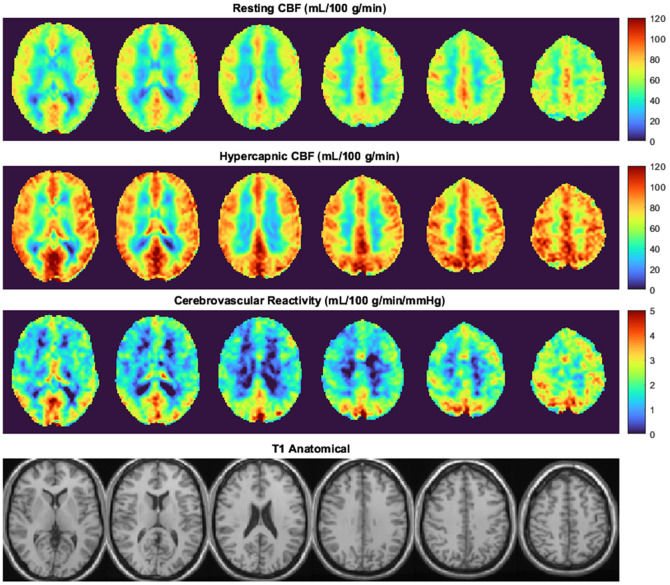
Group average (n=12) ASL images acquired at baseline (normocapnia) and hypercapnia. The voxel-wise map of cerebrovascular reactivity was calculated as the perfusion change from normocapnia to hypercapnia, divided by 9 mmHg. The bottom row shows corresponding T1-weighted MR images for anatomical reference.

Figure 3 presents absolute CBF images acquired with pCASL during normocapnia and hypercapnia (n=12). Overall, ${\mathrm{CBF}}_{\mathrm{ASL}}$ increased by 38±9% (68±11  mL/100  g/min; p<0.01 versus baseline) during hypercapnia (9±1  mmHg $P_{\mathrm{ET}}{\mathrm{CO}}_{2}$). The average ${\mathrm{CVR}}_{\mathrm{CO}2}$ was 4.1±1.0%/mmHg, with no difference between males and females (4±1 versus 4±0%/mmHg; p=0.42).

### 4.3 ΔC_Hb_ and ΔC_HbO_ during trNIRS Hypercapnia Protocol

**Fig. 4:**
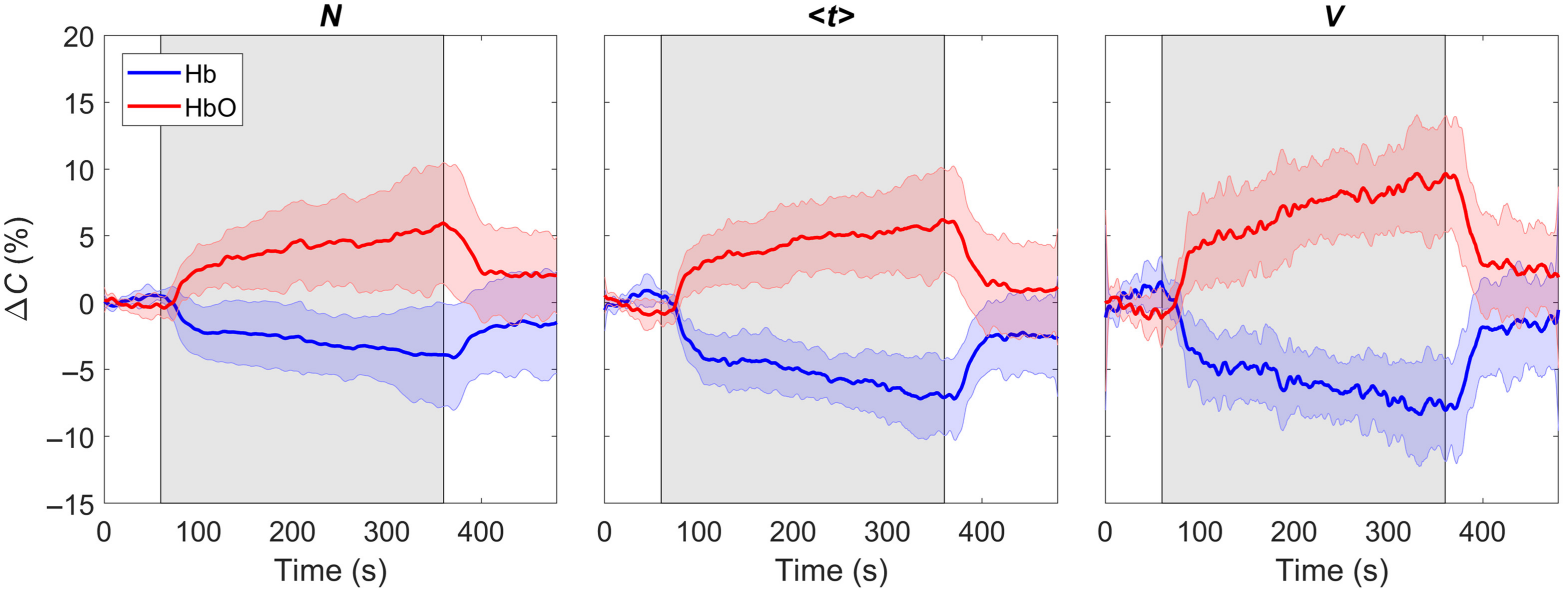
Average time courses of concentration changes in oxyhemoglobin $\Delta C_{\mathrm{HbO}}$ and deoxy-hemoglobin $\Delta C_{\mathrm{Hb}}$ as derived from the first three statistical moments (number of photons N, mean time of flight ⟨t⟩, and variance V) throughout hypercapnia protocol ($+9\;\mathrm{mmHg}\;P_{\mathrm{ET}}{\mathrm{CO}}_{2}$; grey rectangle). Shading around each line represents the standard deviation (n=9).

The average time courses of $\Delta C_{\text{HbO}}$ and $\Delta C_{\mathrm{Hb}}$ during hypercapnia are illustrated in Fig. 4. These time courses were derived from moment analysis of the trNIRS data. Significant interaction effects (moment-by-time) revealed differences between the moments during hypercapnia for both $\Delta C_{\mathrm{HbO}}$ and $\Delta C_{\mathrm{Hb}}$ (both p<0.01). For example, the $\Delta C_{\mathrm{HbO}}$ obtained with ΔV was significantly greater than that calculated using ΔN between 240 and 300 s (both p≤0.049). However, there were no differences when comparing Δ⟨t⟩ to ΔN or ΔV (all p≥0.07). The reduction in $\Delta C_{\mathrm{Hb}}$ calculated by Δ⟨t⟩ was greater than for ΔN between 210 and 300 s (all p≤0.04). However, there was no difference between outcomes from Δ⟨t⟩ and ΔV (all p≥0.28). Importantly, the recovery to baseline was similar between all moments (all p≥0.28). $\Delta \mu_{a}$ increased by 4.57±2.26% during hypercapnia.

### 4.4 CBF_*ASL*_ Versus BFi during ASL Hypercapnia Protocol

**Fig. 5:**
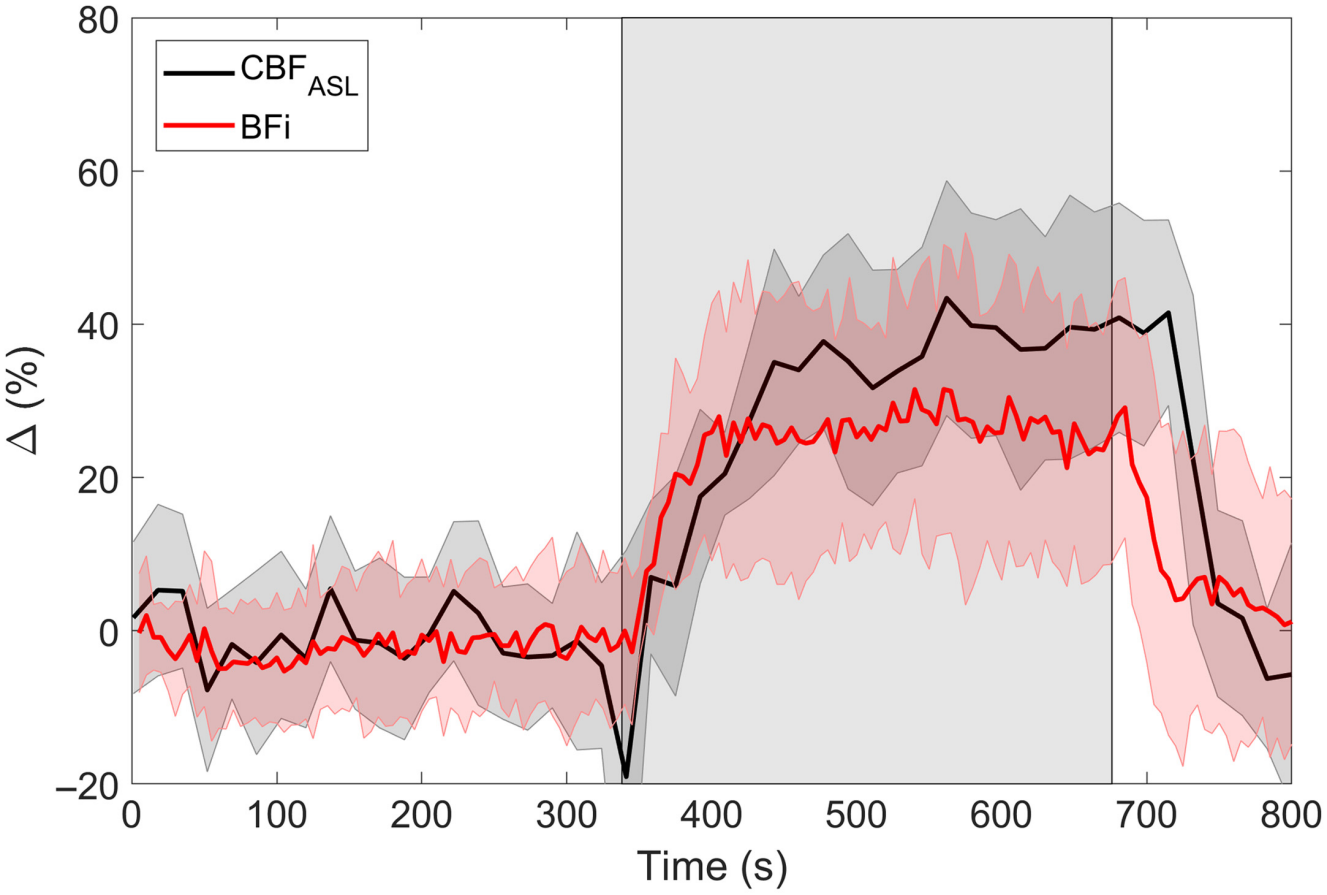
Average time courses of cerebral blood flow calculated using arterial spin labeling (${\mathrm{CBF}}_{\mathrm{ASL}}$) and DCS (BFi). Shading around each line represents the standard deviation (n=12). The grey rectangle indicates the hypercapnia period. During steady-state hypercapnia, ${\mathrm{CBF}}_{\mathrm{ASL}}$ increased by 38±9% and BFi increased by 27±12%.

**Fig. 6:**
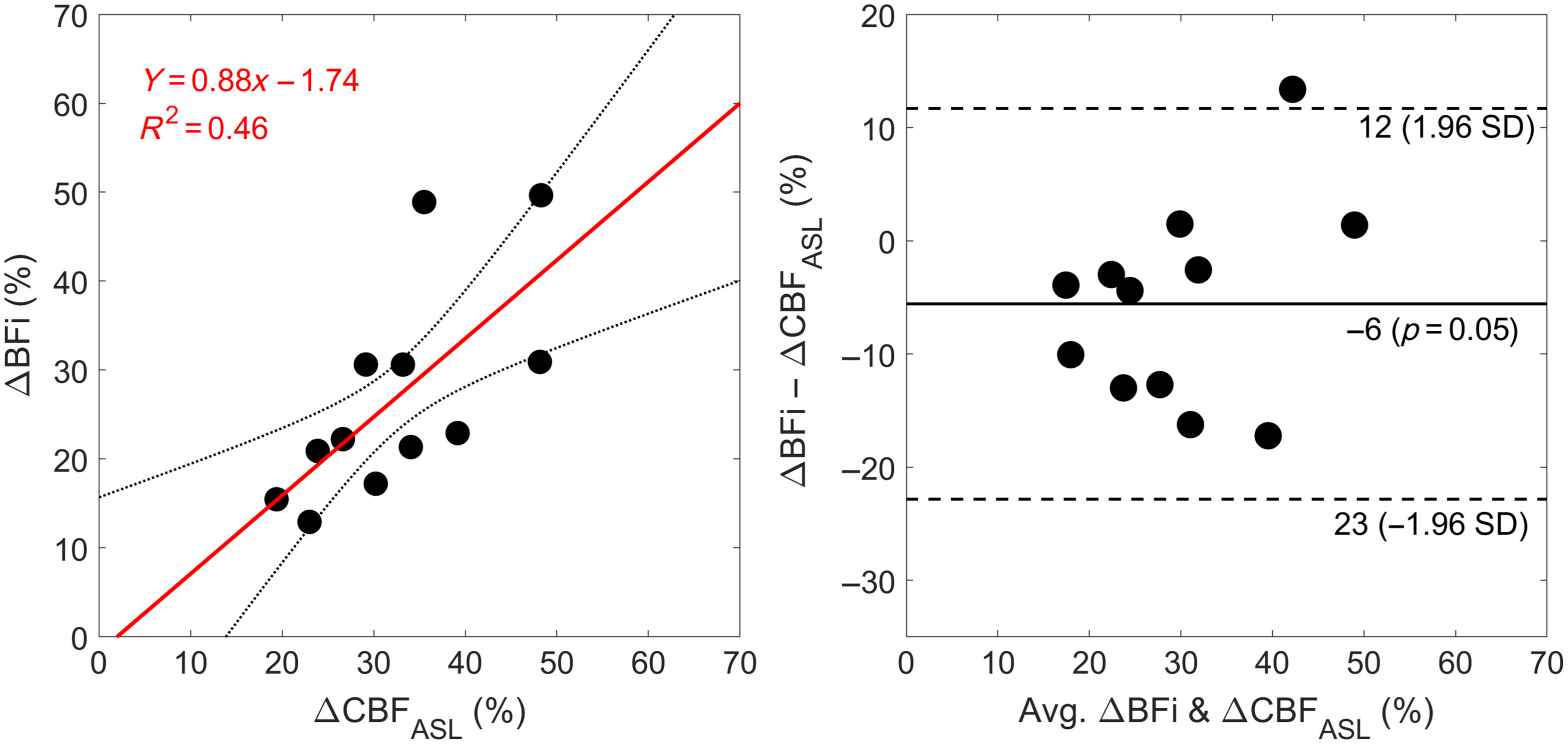
Regression and Bland–Altman plots comparing the hypercapnic CBF change measured by arterial spin labeling (${\mathrm{CBF}}_{\mathrm{ASL}}$) and DCS (BFi) (n=12). The mean difference between the two methods is indicated by the solid black line, which was bound by a 95% confidence interval indicated by the dashed black lines.

Figure 5 presents the average relative change CBF during hypercapnia as measured by ASL (grey matter) and DCS. BFi increased by 27±12% (p<0.01 versus baseline), which was not significantly different from ${\mathrm{CBF}}_{\mathrm{ASL}}$ (p=0.05). There was a strong positive and significant correlation between the change in ${\mathrm{CBF}}_{\mathrm{ASL}}$ and BFi (r=0.68, p=0.01; Fig. 6), with a slope of 0.88 that was not significantly different from 1.00 (p=0.68) and an intercept that was significantly lower than 0.00 (p=0.04). BFi slightly underestimated the increase in CBF measured by ASL, as demonstrated by a mean bias of −5.6% with moderately wide limits of agreement [95% CI: −23, 12] and no evident trend or inconsistent variability.

### 4.5 CBF_*ASL*_ Versus CBF_*DCS*_

**Fig. 7:**
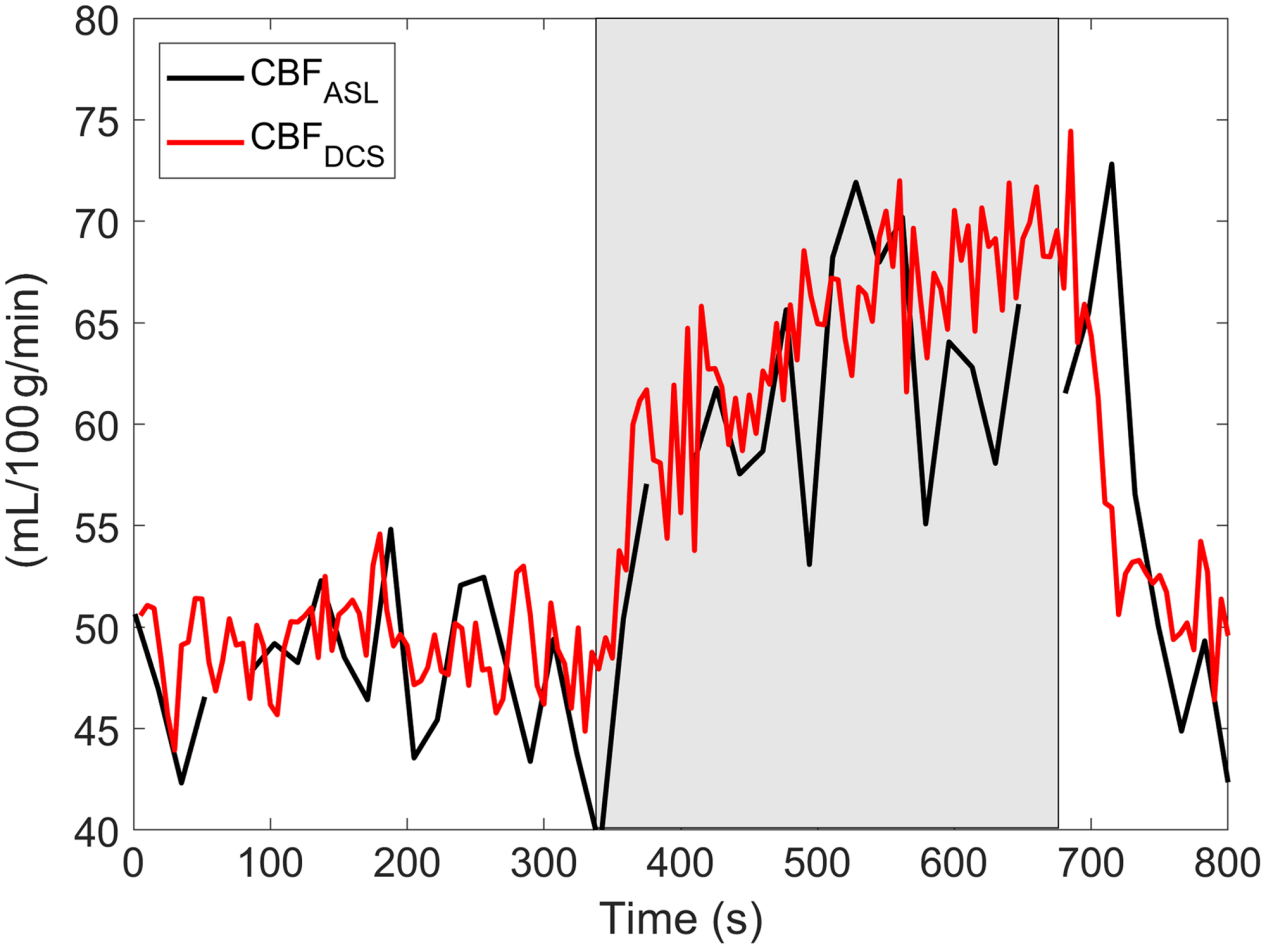
Exemplar CBF time series from one participant from DCS and ASL. For the former, baseline CBF measured by DHC-NIRS was used to scale the DCS data. The hypercapnia period (+9  mmHg) is indicated by the grey rectangle.

**Fig. 8:**
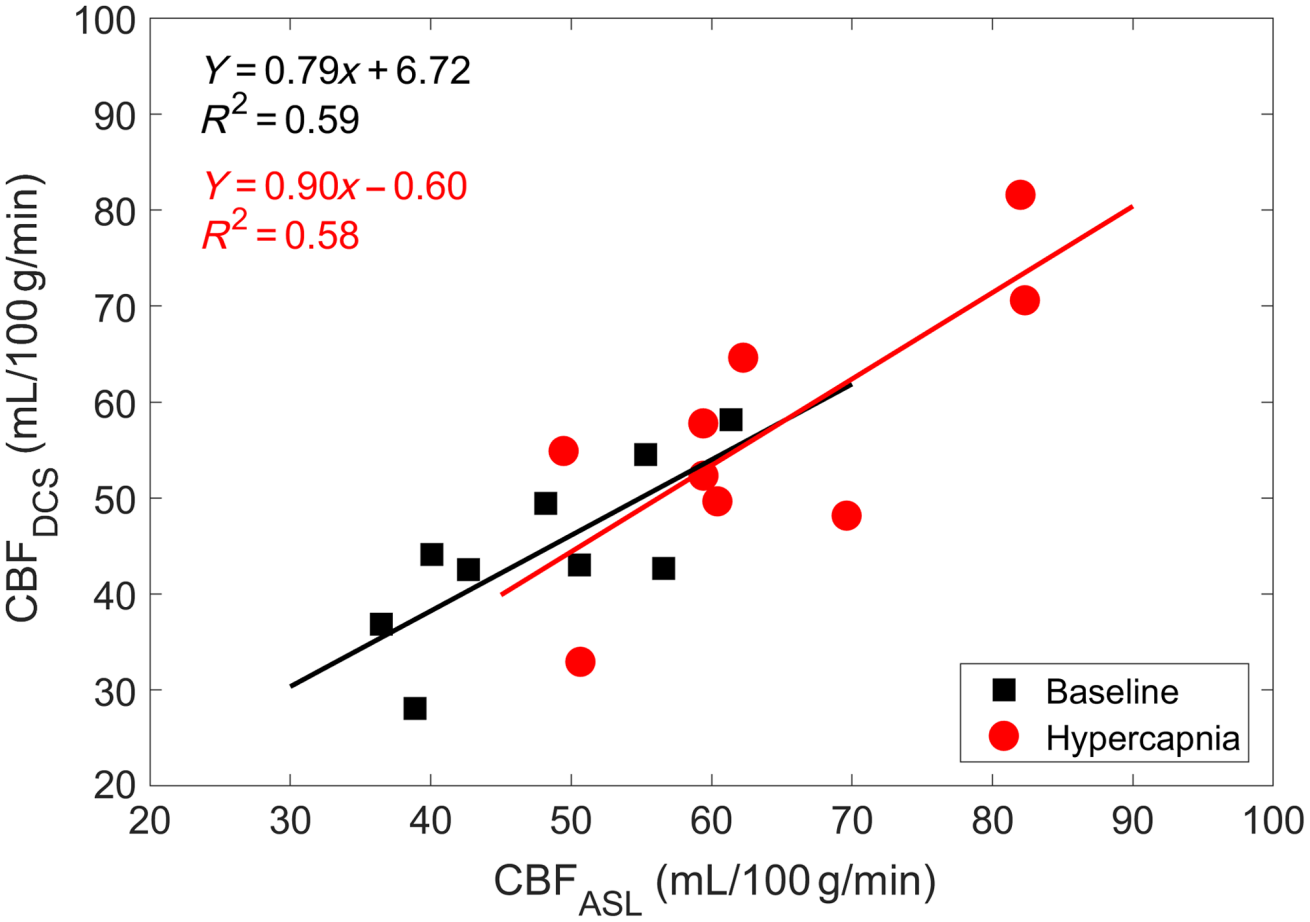
Regression plot comparing CBF values from ASL (${\mathrm{CBF}}_{\mathrm{ASL}}$) and calibrated BFi (${\mathrm{CBF}}_{\mathrm{DCS}}$) during normocapnic (baseline) and hypercapnia (n=9).

Baseline CBF from DHC-NIRS was used to convert the relative BFi time series from DCS into CBF units (i.e., ${\mathrm{CBF}}_{\mathrm{DCS}}$). An exemplary tracing of absolute CBF at rest and during hypercapnia is illustrated in Fig. 7. Moreover, applying this conversion to baseline and hypercapnia BFi values resulted in CBF values that were moderately strong and positively associated with ${\mathrm{CBF}}_{\mathrm{ASL}}$ (both p=0.02; Fig. 8).

## 5 Discussion

The current study demonstrated the accuracy of a non-invasive, all-optics approach for accurately quantifying absolute CBF at rest and during hypercapnia. The main objectives were to investigate the utility of a bolus-tracking NIRS method that involved a brief period of hypoxia to quantify CBF and to investigate the accuracy and sensitivity of calibrated DCS to changes in cerebral perfusion. These outcomes were demonstrated by moderately strong correlations during normocapnia (slope=0.79 and $R^{2}=0.59$) and hypercapnia (slope=0.90 and $R^{2}=0.58$) between CBF values from calibrated DCS and ASL over a range from 34 to 85  mL/100  g/min (Fig. 8). This good agreement was confirmed by Bland–Altman analysis of baseline CBF, which revealed a small bias between the DHC-NIRS and ASL of approximately −3  mL/100  g/min (Fig. 2). These results demonstrate the feasibility of a trNIRS/DCS approach that can both quantify CBF and provide continuous perfusion monitoring.

A recent review of DCS by Carp et al.^4^ called for researchers to validate calibrated DCS perfusion values in clinician-familiar units of flow, with the goal of tracking absolute CBF at the bedside. The current study provides a feasible, rapid, and inexpensive means of performing DCS calibration using NIRS, which is frequently combined with DCS to measure tissue optical properties and ${\mathrm{StO}}_{2}$. The optical dye ICG has been previously proposed for this purpose,^18^^–^^20^ but the use of an exogenous agent complicates the procedure. In contrast, transient hypoxia is safe for healthy and most clinical populations. Previous research on healthy individuals reported no distress at the level of hypoxia used in the current study,^25^^,^^26^^,^^43^ and this condition does not result in noticeable changes in respiratory rate and heart rate.^42^^,^^44^ In fact, rebreathing tests at $P_{\mathrm{ET}}O_{2}$ of 40 and 50 mmHg are commonly used to evaluate respiratory chemoreflexes,^45^ including in patients with heart failure.^46^ Clearly, any clinical application would require careful evaluation for suitability, in particular patients with low ${\mathrm{SaO}}_{2}$ readings. Although the current study used a computer-controlled gas delivery system to induce hypoxia and record the decrease in $P_{\mathrm{ET}}O_{2}$, this protocol could be performed with less expensive technologies: an off-the-shelf pulse oximeter to record ${\mathrm{SaO}}_{2}$^47^ and an inexpensive Douglas bag connected to an $N_{2}$ tank to induce transient hypoxia.^26^ In a previous ICG study,^21^ the correlation with ASL was stronger ($R^{2}=0.88$ compared with $R^{2}=0.59$ in Fig. 2). This difference can be partly explained by the wider perfusion range in the previous study (∼80  mL/100  g/min). In the current study, DHC-NIRS was only applied at baseline because DCS was used to measure the hypercapnic response, which limited the range to ∼30  mL/100  g/min. Another contributing factor is likely the lower contrast-to-noise ratio (CNR) of transient hypoxia compared with a bolus of ICG, which is a strong light absorber in the near-infrared range. The advantage of DHC-NIRS is that the hypoxia protocol can be easily repeated to compensate for the lower CNR.

Using a sudden change in ${\mathrm{SaO}}_{2}$ as a flow tracer was first employed by Edwards et al.^23^ to measure CBF in newborn infants.^23^ Although the method proved accurate, the SNR was low as CBF is determined from the Fick principle, assuming that the deoxygenated bolus does not reach the venous end of the vascular system. This assumption is only satisfied for short durations. In the adult brain, the limit would be ∼5 to 10 s for CBF of 50  mL/100  g/min and a blood volume less than 5  mL/100  g (i.e., mean transit time ∼6  s). Explicitly modeling the transit of deoxygenated hemoglobin through the microvasculature [Eqs. (3) and (4)] overcomes this limitation and enables the whole hypoxia bolus to be used in the analysis (Fig. 1). The application of DHC-NIRS to adults also benefitted from the enhanced depth sensitivity provided by trNIRS. Of the three statistical moments, the mean time of flight was chosen as a balance between CBF sensitivity and SNR, as previously demonstrated by comparing ICG contrast-enhanced curves measured before and after suppressing scalp blood flow.^27^

In addition to measuring baseline CBF, a positive and significant correlation was found between relative BFi and ${\mathrm{CBF}}_{\mathrm{ASL}}$ during hypercapnia (Fig. 6). These BFi values were calculated using the semi-homogeneous model, which is clearly an oversimplification when modeling blood flow in the adult head. We speculate that this approach worked for tracking changes in CBF because the pressure on the optode holder impeded scalp blood flow. Using the optode holder to exert pressure on the scalp has been noted in other studies.^30^^–^^32^ Evidence of its efficacy is provided by the similarly in the hypercapnic $\Delta C_{\mathrm{HbO}}$ and $\Delta C_{\mathrm{Hb}}$ time courses derived from the three moments (Fig. 4). In contrast, we previously observed clear differences between the hemoglobin time courses derived from ΔN, which is most sensitive to the extracerebral layer, and those derived from Δ⟨t⟩ or ΔV, which have greater sensitivity to the brain.^27^ This was interpreted as reflecting differences between extracerebral and cerebral flow responses to hypercapnia. The extracerebral signal appeared sluggish and did not return to baseline upon cessation of ${\mathrm{CO}}_{2}$ inhalation, whereas the cerebral component responded quickly to changes in ${\mathrm{CO}}_{2}$ as expected, considering the cerebral vasculature is very sensitive to ${\mathrm{CO}}_{2}$.^28^ Although we provide evidence that the optode holder minimized the overall extracerebral contribution to our BFi signal, it remains possible that the heterogeneity of the signal explains BFi underestimating ${\mathrm{CBF}}_{\mathrm{ASL}}$ by ∼10% (Fig. 5).

Although the semi-homogeneous model worked well in the current study for tracking BFi changes that reflected CBF, this simplified approach could fail if there were substantial changes in scalp blood flow. In clinical applications, for instance, acute effects of medications or disease complications, such as systemic hypotension, would alter systemic physiology, leading to changes in extracerebral perfusion. Further work is required to explore the utility of short source-detector separation measurements to track scalp blood flow and the application of layered models to separate the flow components. These approaches are more challenging as obtaining reliable blood flow estimates is difficult given the number of unknown parameters in more complex models.^9^^,^^14^^,^^15^

There are a number of potential limitations to the current study. First, only DCS data were acquired as part of the MRI protocol, even though the trNIRS/DCS system was designed to acquire data simultaneously.^28^^,^^33^^–^^35^ This is because the long fibers required to reach the scanner (typically 8 m) degrade the accuracy of the optical property measurements due to the substantial broadening of the IRF.^21^ As a consequence, the analysis of the DCS time series did not incorporate temporal changes in $\mu_{a}$. However, the maximum increase in $\mu_{a}$ during hypercapnia was of the order of <10%, which is predicted to cause an error in BFi of less than 4%.^18^ Another source for the disparity between ${\mathrm{CBF}}_{\mathrm{ASL}}$ and ${\mathrm{CBF}}_{\mathrm{DCS}}$ (Fig. 8) could be the time and transition between the MRI and hospital bed protocols (i.e., ASL hypercapnia protocol versus DHC protocol). For example, changes in baseline CBF would impact the calibration of ${\mathrm{CBF}}_{\mathrm{DCS}}$. A third limitation was that the data presented were collected from young, healthy individuals. Larger studies are required to assess the reliability and validity of this approach in a wider sample, particularly in patients with cerebrovascular pathologies. As Sayin et al.^25^ discussed, the transient hypoxia methodology is in its early stages and requires further optimization of the protocol and analysis. Although we extended their approach using depth-enhanced NIRS, there was still variability in our dataset. We anticipate improved accuracy of our method with further models of depth-enhanced optics.

## 6 Conclusion

In summary, this study demonstrates a non-invasive, all-optics approach to measuring cerebral blood flow in humans. The good agreement, when compared with CBF measurements from ASL, suggests that this method could provide quantitative and continuous CBF data at the bedside of critically ill patients.

## Data Availability

The datasets generated during and/or analyzed during the current study are available from the corresponding author upon reasonable request.
